# Prevalence and Determinants of Elevated Urinary Albumin-Creatinine Ratio Among Patients With Type 2 Diabetes Mellitus: A Real-World Study From South Punjab, Pakistan

**DOI:** 10.7759/cureus.113922

**Published:** 2026-08-04

**Authors:** Qazi Masroor Ali, Raheel Khan, Fahad Qaisar, Sadaf Shafique, Ali Imran, Aleena Masroor

**Affiliations:** 1 Medicine, Ava Serene Hospital, Bahawalpur, PAK; 2 Medicine, Quaid-e-Azam Medical College, Bahawalpur, PAK; 3 Medicine, Bahawal Victoria Hospital, Quaid-e-Azam Medical College, Bahawalpur, PAK; 4 Pathology, Quaid-e-Azam Medical College, Bahawalpur, PAK; 5 Medicine, Cholistan Institute of Medical Sciences, Bahawalpur, PAK; 6 Medicine, Bahawal Victoria Hospital, Bahawalpur, PAK

**Keywords:** albumin, albuminuria, ankle brachial index, creatinine, pakistan, type-2 diabetes mellitus

## Abstract

Objective

This study aimed to determine the prevalence and determinants of elevated urinary albumin-creatinine ratio (UACR) among type 2 diabetes mellitus (T2DM) patients.

Methodology

This analytical cross-sectional study was conducted from January to June 2025 at the Medicine Outpatient Department of Aleena Hospital, Bahawalpur, South Punjab, Pakistan. Adults with T2DM (duration ≥ 6 months) were enrolled, and relevant sociodemographic and clinical data were gathered. Elevated UACR was defined as UACR ≥ 30 mg/g. Data were analyzed using IBM SPSS Statistics, version 26.0 (IBM Corp., Armonk, NY). To determine independent predictors of elevated UACR, multivariate logistic regression analysis was performed, with adjusted odds ratios (aORs) and 95% confidence intervals (CIs) reported. A p-value < 0.05 was considered statistically significant.

Results

Of 495 T2DM patients, 260 (52.5%) were male, and the median age was 50.0 (42.0-56.0) years. The median BMI was 28.7 (25.4-32.0) kg/m², with 393 (79.4%) having a BMI ≥ 25 kg/m². Hypertension was present in 229 (46.3%) patients, and a family history of diabetes was present in 308 (62.2%) patients, whereas the median diabetes duration was 5.0 (3.0-10.0) years. Microalbuminuria (UACR between 30.0-299.0 mg/g) was found in 167 (33.7%) patients, and macroalbuminuria (UACR ≥ 300 mg/g) was found in 29 (5.9%) patients, giving an elevated UACR level in 196 (39.6%) patients. In the multivariable model, diabetes duration (aOR: 1.1 per year, 95% CI: 1.0-1.1, p = 0.010) and ankle-brachial index (ABI) values of 0.9-1.3 (aOR: 3.2, 95% CI: 1.5-6.9, p = 0.002) and > 1.3 (aOR: 3.6, 95% CI: 1.4-9.1, p = 0.007) remained significant predictors of elevated UACR.

Conclusions

Elevated UACR is very common among patients with T2DM in South Punjab. The critical independent factors include longer diabetes duration and abnormal ABI, which support the importance of regular screening for cardiovascular disease and peripheral vascular disease, as well as prompt screening for diabetic kidney disease in outpatient care, especially in resource-limited settings.

## Introduction

Type 2 diabetes mellitus (T2DM) is a major global health challenge and a leading cause of chronic kidney disease (CKD), cardiovascular events, and premature mortality. The International Diabetes Federation (IDF) estimated that 536.6 million adults (10.5%) were living with diabetes in 2021, with projections rising to 783.2 million (12.2%) by 2045 [1]. Pakistan is among the countries with the highest disease burden, with recent IDF data from 2024 indicating that approximately 34.5 million adults are affected by diabetes, corresponding to an adult prevalence of 31.4%, the highest reported prevalence globally among individuals aged 20-79 years [2].

As the number of people with T2DM increases, diabetes-related microvascular complications are expected to rise in parallel. Albuminuria is a key marker for the early detection and staging of diabetic kidney disease (DKD) [3]. Kidney Disease: Improving Global Outcomes (KDIGO) guidelines classify albuminuria as A1 (urinary albumin-creatinine ratio (UACR) < 30 mg/g), A2 (30-299 mg/g), and A3 (≥ 300 mg/g), and recommend that CKD staging in diabetes incorporate both estimated glomerular filtration rate (eGFR) and albuminuria [4]. Elevated UACR reflects glomerular damage and is independently associated with faster CKD progression, cardiovascular events, and all-cause mortality, even when eGFR is relatively preserved [5]. Accordingly, annual UACR screening is recommended for all patients with T2DM to enable the timely initiation of reno-protective strategies [6,7].

A local multicentre study from Karachi reported microalbuminuria in 34% of T2DM subjects, with significant associations with age, diastolic blood pressure, serum low-density lipoprotein (LDL) cholesterol, and diabetic retinopathy [8]. More recent data from Sargodha showed that 53% of T2DM patients had some degree of albuminuria, including 37% with microalbuminuria and 16% with macroalbuminuria, and that albuminuria was strongly related to longer duration of diabetes, hypertension, obesity, physical inactivity, and smoking [9]. These findings underscore that early renal involvement is common in Pakistani T2DM populations and is driven largely by modifiable cardiometabolic risk factors.

However, most Pakistani studies on UACR and DKD have been conducted in large public or teaching hospitals and may not reflect patients managed in private outpatient settings, particularly in South Punjab, Pakistan, where healthcare access, health-seeking behaviors, and risk profiles may differ. Recent regional work from South Punjab, Pakistan, illustrates the high burden of end-organ complications in such real-world settings, as a 53.8% prevalence of hepatic steatosis was observed among patients with T2DM and obesity, and shorter diabetes duration and hypertension were documented as important correlates [10]. This pattern of clustered metabolic complications suggests that renal involvement is also likely to be common in similar outpatient T2DM cohorts, but local data are scarce [11].

The present study was conducted at a private healthcare outpatient facility in South Punjab, Pakistan, to determine the prevalence and determinants of elevated UACR among patients with T2DM. By combining UACR measurements with detailed sociodemographic and clinical information, this study aims to provide region-specific evidence to support risk-stratified screening and early reno-protective interventions in a high-risk yet understudied segment of the Pakistani diabetic population. South Punjab includes a large rural and semi-urban catchment population where health-seeking behavior, literacy, household income, affordability of long-term diabetes care, and access to screening for chronic complications may differ from those in more urbanized regions of Pakistan.

## Materials and methods

This analytical cross-sectional study was conducted at the Outpatient Department of Medicine, Aleena Hospital, Bahawalpur, Pakistan, a private healthcare outpatient facility in South Punjab. It provides diabetology and general medical services to urban and affiliated rural communities. The study was conducted from January 2025 to June 2025. Inclusion criteria included patients with T2DM aged 18 years or older who attended the outpatient department during the predefined study period. T2DM diagnosis was documented in medical records based on a prior diagnosis and/or the use of glucose-lowering medication, with a known diabetes duration of at least six months. A diabetes duration of at least six months was chosen because it is generally considered sufficient to ensure that participants had passed the initial acute post-diagnostic phase, during which lifestyle adjustments, acute stress, and frequent therapeutic titrations commonly occur. This threshold allows patients' glycemic control (such as HbA1c stability) and medication regimens to reach a steady maintenance state, thereby reducing confounding variables related to newly diagnosed disease and providing a more homogeneous study population. 

Exclusion criteria included individuals with type 1 diabetes, gestational or secondary diabetes, known non-diabetic kidney disease, current urinary tract infection or macroscopic hematuria, acute febrile illness, or pregnancy. Non-diabetic kidney disease was excluded through a review of available clinical records, physician assessment, history of previously diagnosed renal disease, current symptoms suggestive of urinary tract pathology, and urinalysis findings where available. Patients with known non-diabetic kidney disease, current urinary tract infection, macroscopic hematuria, pregnancy, or acute febrile illness were excluded to reduce the possibility of transient or non-diabetic causes of albuminuria.

A non-probability, consecutive sampling strategy was used, as all patients meeting eligibility criteria and presenting during routine clinic hours were invited to participate. After explanation of the study objectives and procedures in the local language, written informed consent was obtained. A sample size of 345 was calculated based on an anticipated proportion of microalbuminuria of 34% [8], with a 95% confidence level (CI) and a 5% margin of error. For this study, a total of 495 patients fulfilled the inclusion criteria, provided consent, and had complete data available for analysis; therefore, these 495 patients constituted the final analytical sample. The study protocol was approved by the hospital ethics review committee (letter number: HRC/06/2024, dated October 16, 2024), and all procedures adhered to the principles of the Declaration of Helsinki, with strict maintenance of patient confidentiality and anonymization of data before analysis.

Two trained medical officers were responsible for screening patients for eligibility, obtaining informed consent, conducting structured interviews, and performing physical examinations. A nursing staff member and a clinic assistant, working under the supervision of the medical officers, performed anthropometric measurements and assisted with blood pressure and ankle-brachial index (ABI) assessments. Certified laboratory technologists from the clinic’s affiliated laboratory collected and processed blood and urine samples, while a data entry operator entered anonymized data into the electronic database.

Sociodemographic and clinical information was documented at the time of enrollment. Hypertension was defined as a documented diagnosis of hypertension in the medical record, current use of antihypertensive medication, or a measured blood pressure ≥ 140/90 mmHg on the day of assessment. BMI was categorized according to Asian-specific cut-offs as underweight (< 18.5 kg/m²), normal weight (18.5-22.9 kg/m²), overweight (23.0-24.9 kg/m²), and obese (≥ 25.0 kg/m²). Peripheral vascular status was assessed using the ankle-brachial index (ABI). The same medical officers performed all ABI measurements following a standard protocol to reduce inter-observer variability.

After the patient had rested in the supine position for at least 10 minutes, systolic blood pressure was measured at the brachial artery in both arms using a handheld Doppler device and sphygmomanometer. Systolic pressures were then measured at the dorsalis pedis and/or posterior tibial arteries of both ankles. The right ankle-brachial index (RABI) was calculated as the ratio of the higher of the right dorsalis pedis or posterior tibial systolic pressure to the higher brachial systolic pressure; similarly, the left ankle-brachial index (LABI) was calculated using the left ankle systolic pressure. ABI values were recorded as continuous variables and, where appropriate, categorized (e.g., < 0.9, 0.9-1.3, and > 1.3) for analysis [12]. Biochemical measurements were obtained after an overnight fast of at least eight hours, whenever feasible.

Urine albumin and creatinine were measured from a spot midstream urine sample collected on the same day as the clinic visit, preferably in the morning. Patients were instructed on proper clean-catch midstream collection. Samples were transported promptly to the laboratory and analyzed within the recommended time frame. Urinary albumin concentration (mg/L) was determined using an immunoturbidimetric assay, while urinary creatinine concentration (mg/dL) was measured using an enzymatic or Jaffe method, according to the laboratory’s standard operating procedures. The UACR was calculated by dividing urinary albumin by urinary creatinine (mg/g). For the main analyses, albuminuria categories were defined as follows: no albuminuria, UACR < 30 mg/g; microalbuminuria, UACR 30-299 mg/g; and macroalbuminuria, UACR ≥ 300 mg/g. Albuminuria for this study was defined as UACR ≥30 mg/g, representing the combination of moderately and severely increased albuminuria [13]. Urinary albumin and creatinine assays were performed in the same institutional laboratory using standard operating procedures. Internal quality control procedures were followed according to the laboratory protocol to ensure the consistency and reliability of UACR measurements. The resulting UACR value was used as the primary outcome variable of the study.

The medical officers involved in data collection were trained to conduct interviews in a uniform and non-leading manner. To reduce researcher bias, the same structured sequence of questions was followed for all participants, responses were recorded directly on the study proforma, and objective clinical and laboratory parameters were measured using standardized procedures. For participants with limited literacy, the study information and consent form were explained verbally in the simple local language. The objectives of the study, required procedures, confidentiality of data, voluntary participation, and right to refuse or withdraw were explained before enrollment. Participants were encouraged to ask questions, and consent was obtained only after adequate understanding was confirmed. For participants unable to read or sign, the consent form was read aloud, and a thumb impression was obtained in the presence of an attendant/witness where applicable.

Data were entered and analyzed using IBM SPSS Statistics, version 26.0 (IBM Corp., Armonk, NY). Continuous variables were summarized as means and standard deviations (SDs) or medians and interquartile ranges (IQRs), depending on distribution, whereas categorical variables were presented as frequencies and percentages. Categorical data were compared using chi-square or Fisher’s exact tests, and independent t-tests or Mann-Whitney U tests, as well as analysis of variance or Kruskal-Wallis tests, were performed for continuous variables. To identify independent determinants of elevated UACR, a multivariable logistic regression model was constructed, including variables with p < 0.10 in univariable analyses. Adjusted odds ratios (aORs) with 95% CIs were reported. For the multivariable logistic regression model, model fit was assessed using the Hosmer-Lemeshow goodness-of-fit test, and the overall classification accuracy was recorded from the classification table. Multicollinearity among independent variables was assessed using tolerance and variance inflation factor values, with tolerance < 0.10 or variance inflation factor > 10 considered suggestive of significant multicollinearity. For all inferential statistics, p < 0.05 was considered statistically significant.

## Results

Of the total 495 patients with T2DM, 260 (52.5%) were male, and the median age was 50.0 (42.0-56.0) years (range, 18-75 years). Educational status was reported as illiterate in 238 (48.1%) patients. Rural residence was reported by 370 (74.7%) patients. The median BMI was 28.7 (25.4-32.0) kg/m², and 393 (79.4%) patients had a BMI ≥ 25 kg/m². Hypertension was present in 229 (46.3%) patients, while 308 (62.2%) patients reported a family history of diabetes. The median duration of diabetes was 5.0 (3.0-10.0) years, with a range of 1-29 years. Microalbuminuria was identified in 167 (33.7%) patients, while 29 (5.9%) had macroalbuminuria. Lower monthly family income (p = 0.008), higher BMI category (p = 0.001), and longer diabetes duration (p = 0.029) were significantly associated with albuminuria (Table 1).

**Table 1:** Association of demographic and clinical variables with albuminuria stratification (N = 495) ^^^Chi-square test; ^#^Fisher’s exact test; ^*^Kruskal-Wallis test P < 0.05 considered statistically significant HCV: hepatitis C virus; HBsAg: hepatitis B surface antigen; IQR: interquartile range; PKR: Pakistani rupees

Variables			No albuminuria (n = 299)	Microalbuminuria (n = 167)	Macroalbuminuria (n = 29)	Test statistics	P-value
Gender, n (%)		Male	159 (53.2%)	87 (52.1%)	14 (48.3%)	0.273	0.872^^^
		Female	140 (46.8%)	80 (47.9%)	15 (51.7%)		
Age, years, median (IQR)			50.0 (43.0-58.0)	52.5 (47.0-56.8)	52.0 (45.8-61.0)	0.824	0.662^*^
Education, n (%)		Illiterate	131 (43.8%)	91 (54.5%)	16 (55.2%)	5.515	0.063^^^
		Literate	168 (56.2%)	76 (45.5%)	13 (44.8%)		
Residence, n (%)		Rural	229 (76.6%)	120 (71.9%)	21 (72.4%)	1.360	0.507^^^
		Urban	70 (23.4%)	47 (28.1%)	8 (27.6%)		
Monthly family income, PKR, n (%)		< 30,000	103 (34.4%)	81 (48.5%)	7 (24.1%)	13.861	0.008^^^
		30,000 to 70,000	186 (62.2%)	84 (50.3%)	20 (69.0%)		
		> 70,000	10 (3.3%)	2 (1.2%)	2 (6.9%)		
Body mass index, kg/m^2^, n (%)		Underweight	6 (2.0%)	4 (2.4%)	1 (3.4%)	21.714	0.001^^^
		Normal	22 (7.4%)	24 (14.4%)	2 (6.9%)		
		Overweight	20 (6.7%)	15 (9.0%)	8 (27.6%)		
		Obesity	251 (83.9%)	124 (74.3%)	18 (62.1%)		
History of smoking, n (%)			6 (2.0%)	6 (3.6%)	-	1.905	0.386^^^
Hypertension, n (%)			139 (46.5%)	74 (44.3%)	16 (55.2%)	1.188	0.552^^^
Family history of diabetes, n (%)			187 (62.5%)	101 (60.5%)	20 (69.0%)	0.790	0.674^^^
Duration of diabetes, years, median (IQR)			5.0 (2.0-8.0)	5.0 (2.3-11.5)	7.5 (4.5-13.3)	7.061	0.029^*^
HBsAg-positive, n (%)			2 (0.1%)	4 (2.4%)	0 (0%)	3.045	0.218^#^
Anti-HCV-positive, n (%)			32 (10.7%)	19 (11.3%)	3 (10.3%)	0.060	0.970^#^
Liver cirrhosis, n (%)			10 (3.3%)	9 (5.4%)	1 (3.4%)	1.183	0.553^^^
Ankle-brachial index, n (%)	< 0.9		60 (20.1%)	19 (11.4%)	2 (6.9%)	8.515	0.074^^^
	0.9-1.3		202 (67.6%)	121 (72.5%)	22 (75.9%)		
	> 1.3		37 (12.4%)	27 (16.2%)	5 (17.2%)		
HbA1c, %, median (IQR)			8.9 (7.2-10.0)	9.1 (7.7-10.3)	8.5 (7.2-12.7)	0.977	0.614^*^

When patients were stratified according to UACR status, 196 (39.6%) had elevated UACR. Illiteracy (p = 0.019), higher BMI (p = 0.020), longer duration of diabetes (p = 0.008), and higher ABI (p = 0.017) were significantly associated with elevated UACR (Table 2).

**Table 2:** Association of elevated UACR with demographic and clinical variables in patients with T2DM (N = 495) ^*^Chi-quare test; ^^^Mann-Whitney U test; ^^^^Fisher’s exact test P < 0.05 considered statistically significant UACR: urinary albumin-creatinine ratio; T2DM; type 2 diabetes mellitus; IQR: interquartile range; HCV: hepatitis C virus; HBsAg: hepatitis B surface antigen; PKR: Pakistani rupees

Variables		UACR not elevated (n = 299)	UACR elevated (n = 196)	Test statistics	P-value
Gender, n (%)	Male	159 (53.2%)	101 (51.5%)	0.129	0.720^*^
	Female	140 (46.8%)	95 (48.5%)		
Age, years, median (IQR)		50.0 (43.0-58.0)	52.5 (46.5-56.8)	0.757	0.449^^^
Education, n (%)	Illiterate	131 (43.8%)	107 (54.6%)	5.510	0.019^*^
	Literate	168 (56.2%)	89 (45.4%)		
Residence, n (%)	Rural	229 (76.6%)	141 (71.9%)	1.356	0.244^*^
	Urban	70 (23.4%)	55 (28.1%)		
Monthly family income (PKR), n (%)	< 30,000	103 (34.4%)	88 (44.9%)	5.752	0.056^*^
	30,000 to 70,000	186 (62.2%)	104 (53.1%)		
	> 70,000	10 (3.3%)	4 (2.0%)		
Body mass index, kg/m^2^, n (%)	Underweight	6 (2.0%)	5 (2.6%)	9.860	0.020^*^
	Normal	22 (7.4%)	26 (13.3%)		
	Overweight	20 (6.7%)	23 (11.7%)		
	Obesity	251 (83.9%)	142 (72.4%)		
History of smoking, n (%)		6 (2.0%)	6 (3.1%)	0.557	0.456^*^
Hypertension, n (%)		139 (46.5%)	90 (45.9%)	0.015	0.901^*^
Family history of diabetes, n (%)		187 (62.5%)	121 (61.7%)	0.033	0.856^*^
Duration of diabetes, years, median (IQR)		5.0 (2.0-8.0)	5.5 (3.0-11.5)	2.653	0.008^^^
HBsAg-positive, n (%)		2 (66.9%)	4 (2.0%)	0.861	0.173^^^^
Anti-HCV-positive, n (%)		32 (10.7%)	22 (11.2%)	0.033	0.855^*^
Liver cirrhosis, n (%)		10 (3.3%)	10 (5.1%)	0.943	0.331^*^
Ankle-brachial index, n (%)	< 0.9	60 (20.1%0	21 (10.7%)	8.151	0.017^*^
	0.9-1.3	202 (67.6%)	143 (73.0%)		
	> 1.3	37 (12.4%)	32 (16.3%)		
HbA1c, %, median (IQR)		8.9 (7.2-10.0)	9.0 (7.7-10.8)	0.966	0.334^^^

On multivariable binary logistic regression, each additional year of diabetes duration was associated with a 10% increase in the odds of having elevated UACR (aOR: 1.1; 95% CI: 1.0-1.1; p = 0.010). Compared with patients who had an ABI < 0.9, those with an ABI of 0.9-1.3 had 3.2-fold higher odds of elevated UACR (aOR: 3.2; 95% CI: 1.5-6.9; p = 0.002), and those with an ABI > 1.3 had 3.6-fold higher odds (aOR: 3.6; 95% CI: 1.4-9.1; p = 0.007). Educational status and monthly family income did not show statistically significant associations with elevated UACR in the adjusted model (Table 3).

**Table 3:** Binary logistic regression analysis for the predictors of elevated UACR in T2DM patients P < 0.05 considered statistically significant UACR: urinary albumin-creatinine ratio; T2DM; type 2 diabetes mellitus; CI: confidence interval; OR: odds ratio; PKR: Pakistani rupees

Predictors		Adjusted OR	95% CI (lower-upper)	P-value
Education	Illiterate	1.4	0.9-2.2	0.141
	Literate	Reference		
Monthly family income, PKR	< 30,000	2.8	0.7-11.6	0.147
	30,000 to 70,000	1.5	0.4-5.9	0.587
	> 70,000	Reference		
Body mass index, kg/m^2^	Underweight	Reference		
	Normal	2.3	0.5-10.2	0.291
	Overweight	1.5	0.3-6.9	0.596
	Obesity	0.7	0.2-2.9	0.662
Diabetes duration, years		1.1	1.0-1.1	0.010
Ankle-brachial index	< 0.9	Reference		
	0.9-1.3	3.2	1.5-6.9	0.002
	> 1.3	3.6	1.4-9.1	0.007

## Discussion

In this study among T2DM patients visiting a private outpatient healthcare facility in South Punjab, microalbuminuria was identified in 33.7%, and macroalbuminuria in 5.9% of patients. Ahmadani et al. in Karachi described microalbuminuria in 24.2% and macroalbuminuria in 9.1% of adults with T2DM, while Pasko et al. in Tirana reported microalbuminuria in 38.6% and macroalbuminuria in 3.1% of T2DM patients [14,15]. Al-Salman et al. in Bahrain found albuminuria in 27.9% of T2DM patients, with microalbuminuria in 22.0% and macroalbuminuria in 5.8% [16]. Al-Shammakh et al. in Yemen observed microalbuminuria in 49% and macroalbuminuria in 25.5% of diabetic patients [17,18]. These findings suggest that patients with T2DM receiving routine care in a private facility in South Punjab experience a burden of early kidney involvement comparable to that seen in other populations with high cardiometabolic risk.

Lower income and higher BMI were associated with albuminuria on univariable analysis, which aligns with patterns in several international datasets. A study from Baghdad, Iraq, demonstrated significant associations between microalbuminuria and BMI, while Al-Shammakh reported obesity as a risk factor for pathological proteinuria [17,18]. Pasko et al. also found that central adiposity and higher blood pressure were linked with microalbuminuria. In the current multivariable model, BMI categories did not retain independent significance, which might reflect collinearity with other variables such as diabetes duration or blood pressure, or limited power in the underweight reference group [15]. The direction of effect is consistent with the notion that excess adiposity promotes glomerular hyperfiltration and endothelial dysfunction, leading to albumin leakage, but the adjusted estimates suggest that once vascular and glycemic factors are included, BMI alone is a weaker discriminator of risk [19].

The high prevalence of BMI ≥ 25 kg/m² in the present study is clinically important because obesity is closely linked with insulin resistance, hypertension, inflammation, endothelial dysfunction, and renal hemodynamic changes, all of which may contribute to albuminuria in T2DM. In the present analysis, BMI category was associated with elevated UACR on univariable comparison but was not retained as an independent correlate after adjustment. This finding should be interpreted cautiously. One possible explanation is that obesity was highly prevalent in the overall sample, reducing the contrast between comparison groups. Another explanation is that BMI alone may not adequately capture visceral or central adiposity, which may be more strongly related to albuminuria than generalized body mass.

Diabetes duration in this study had a median of five years, and each additional year conferred roughly a 10% higher odds of elevated UACR. Al-Salman et al. in Bahrain identified longer disease duration as an independent predictor of albuminuria [16]. Pasko et al. showed that the odds of microalbuminuria became significantly raised after 16 years of diagnosed diabetes, with 43.7% affected at that time point [15]. Al-Shammakh et al. also found a graded relation between duration categories and pathological proteinuria, with adjusted odds ratios of 5.55 and 8.88 for 5-10 years and beyond 10 years, respectively [18]. These convergent findings across diverse populations reinforce diabetes duration as a simple but powerful marker for targeting kidney disease screening and intensifying cardio-renal prevention.

The observation that higher ABI categories were linked with substantially greater odds of elevated UACR is one of the more distinctive features of this study. Albuminuria is widely regarded as a marker of systemic endothelial injury and microvascular damage, and the parallel between kidney and peripheral vascular involvement is biologically plausible [20,21]. Pijls et al., in a primary care diabetic cohort, reported that albuminuria was independently associated with systolic blood pressure, smoking, glycosuria, and male gender, all factors that also predispose to peripheral artery disease [22]. Le et al. showed that a composite kidney dysfunction index based on eGFR and UACR correlated with 10-year cardiovascular risk, supporting the concept of shared vascular pathways [23]. The present data may extend these findings by linking albuminuria with ABI in an outpatient South Asian cohort, suggesting that simple bedside vascular measurements may help flag patients at elevated risk of early DKD.

Education and monthly family income showed an association with albuminuria on crude comparison, particularly a higher prevalence of elevated UACR in individuals who were illiterate or had lower income. After adjustment for diabetes duration and ABI, these variables were no longer statistically significant. Ali et al. found a significant association between educational level and microalbuminuria in Baghdad, while some other series did not report strong independent effects once clinical factors were taken into account [17]. In this South Punjab population, socioeconomic disadvantage may influence kidney risk indirectly through access to care, late diagnosis, and suboptimal control of blood pressure and glucose, which are partly captured by the clinical covariates entered in the model [24]. Future work that includes direct measures of treatment adherence, dietary quality, and health literacy may clarify pathways through which social position shapes renal outcomes.

The present findings sit alongside extensive evidence that poor glycemic control is associated with urinary albumin loss, although glycated haemoglobin did not emerge as an independent predictor in this model. Qamar et al. observed that higher glycated haemoglobin levels were linked with worse kidney function, including a higher albumin-creatinine ratio in Pakistani adults with T2DM [25]. Bae et al., using national survey data from Korea, identified fasting blood sugar and glycated haemoglobin as significant risk factors for microalbuminuria [26]. Al-Salman et al. also reported that high glycated haemoglobin was a key predictor of albuminuria [16]. The lack of a clear independent effect in the current analysis may relate to relatively narrow variation in glycated haemoglobin within this private clinic cohort or to residual confounding by diabetes duration, since long-standing disease combines cumulative glucose exposure with structural kidney changes that are not fully captured by a single glycated haemoglobin value.

Literacy status and rural residence are important contextual factors in interpreting the present findings. A substantial proportion of patients may have faced barriers related to health literacy, affordability, travel distance, and regular access to diabetes care. Limited literacy can reduce awareness of asymptomatic complications such as albuminuria, impair understanding of long-term glycemic and blood pressure control, and affect adherence to lifestyle advice, medication schedules, and follow-up visits. Rural residence may influence health-seeking behavior through delayed presentation, reliance on episodic care, transportation difficulties, and reduced access to laboratory screening. These factors are particularly relevant for UACR testing because albuminuria is usually clinically silent and may remain undetected unless routine complication screening is performed.

Dietary and socio-cultural context should also be considered while interpreting the present findings. South Punjab shares many broader Punjabi and South Asian dietary practices, including carbohydrate-based staple meals, frequent tea consumption, and the use of fats in traditional foods. However, the region also includes a large rural and semi-urban population in which food choices, meal timing, affordability of healthier diets, family-based eating patterns, and access to regular diabetes counseling may differ from more urbanized settings. Cultural expectations around hospitality, family meals, festive foods, and perceived “strength-giving” foods may also make long-term dietary modification difficult for patients with T2DM. These factors may partly explain the high burden of obesity observed in the present cohort and may influence glycemic control, medication adherence, and uptake of routine complication screening such as UACR testing. However, as detailed dietary intake and socio-cultural practices were not directly measured, their role could not be formally analyzed in this study.

Clinical implications of these results are substantial. A prevalence of albuminuria approaching nearly two-fifths among T2DM patients in an outpatient private setting indicates that systematic screening with UACR is essential even outside tertiary hospitals. Patients with longer diabetes duration and abnormal ABI constitute a high-risk subset in whom repeat urinary albumin testing, intensive blood pressure control, use of renin angiotensin system blockade, and consideration of agents with proven renal benefit such as sodium glucose cotransporter two inhibitors may be particularly important [27]. The observed associations with low income and excess adiposity on unadjusted analyses point to the need for structured lifestyle counselling and better access to affordable medications in lower-income groups to prevent progression to advanced kidney disease and cardiovascular events.

The cross-sectional design precludes inference about temporal sequence, so it cannot be established whether abnormal ABI preceded the onset of albuminuria or arose in parallel. Another limitation of this study was the unavailability of serum creatinine and estimated glomerular filtration rate for all participants. Therefore, renal function could not be assessed alongside UACR, and the study was limited to albuminuria-based screening rather than complete staging of diabetic kidney disease. Longitudinal cohort studies in similar settings that track changes in UACR and vascular indices over time would allow assessment of directionality and causality. Information on some variables, such as diabetes duration and family history, relied partly on patient report, which may introduce recall inaccuracy.

Future research could strengthen validity by confirming diagnosis dates from electronic records and using standardized family history tools. The study was conducted in a single private clinic in South Punjab, which may limit generalizability to public sector facilities or other regions where case mix and treatment patterns differ. Multicentre studies that include both public and private providers across urban and rural areas of Pakistan would offer a more comprehensive national picture. Finally, the model did not incorporate data on lipid profile, antihypertensive medication type, or novel biomarkers such as cystatin C or kidney dysfunction index, which might further refine risk prediction. Future work can expand the panel of clinical and laboratory predictors to build more informative stratification tools.

## Conclusions

Elevated UACR is very common among patients with T2DM in South Punjab. The critical independent factors include longer diabetes duration and abnormal ABI. These findings support early and repeated screening for UACR in primary and secondary care and highlight the intertwined nature of microvascular and macrovascular damage in T2DM. Early identification of kidney involvement in T2DM patients offers an important opportunity to intensify preventive therapy and potentially reduce the burden of CKD and cardiovascular complications in this high-risk population. Routine screening of UACR and ABI in outpatient diabetic clinics may help identify patients at higher risk of diabetic kidney disease and cardiovascular complications.
